# Genetic tools for the redirection of the central carbon flow towards the production of lactate in the human gut bacterium *Phocaeicola (Bacteroides) vulgatus*

**DOI:** 10.1007/s00253-022-11777-6

**Published:** 2022-01-26

**Authors:** Rebecca Lück, Uwe Deppenmeier

**Affiliations:** Institute of Microbiology and Biotechnology, University of Bonn, Meckenheimer Allee 168, 53115 Bonn, Germany

**Keywords:** Microbiota, Human health, *Bacteroidaceae*, Short-chain fatty acids, Carbon flux

## Abstract

**Abstract:**

Species of the genera *Bacteroides* and *Phocaeicola* play an important role in the human colon. The organisms contribute to the degradation of complex heteropolysaccharides to small chain fatty acids, which are in part utilized by the human body. Furthermore, these organisms are involved in the synthesis of vitamins and other bioactive compounds. Of special interest is *Phocaeicola vulgatus*, originally classified as a *Bacteroides* species, due to its abundance in the human intestinal tract and its ability to degrade many plant-derived heteropolysaccharides. We analyzed different tools for the genetic modification of this microorganism, with respect to homologous gene expression of the *ldh* gene encoding a D-lactate dehydrogenase (LDH). Therefore, the *ldh* gene was cloned into the integration vector pMM656 and the shuttle vector pG106 for homologous gene expression in *P. vulgatus*. We determined the *ldh* copy number, transcript abundance, and the enzyme activity of the wild type and the mutants. The strain containing the shuttle vector showed an approx. 1500-fold increase in the *ldh* transcript concentration and an enhanced LDH activity that was about 200-fold higher compared to the parental strain. Overall, the proportion of lactate in the general catabolic carbon flow increased from 2.9% (wild type) to 28.5% in the LDH-overproducing mutant. This approach is a proof of concept, verifying the genetic accessibility of *P. vulgatus* and could form the basis for targeted genetic optimization.

**Key points:**

*• A lactate dehydrogenase was overexpressed in Phocaeicola (Bacteroides) vulgatus.*

*• The ldh transcript abundance and the LDH activity increased sharply in the mutant.*

*• The proportion of lactate in the catabolic carbon flow increased to about 30%.*

**Supplementary Information:**

The online version contains supplementary material available at 10.1007/s00253-022-11777-6.

## Introduction

The human colon contains a versatile and complex microbial flora, and the majority of these organisms proliferate only under anaerobic conditions (Sender et al. 2016; Lu and Imlay 2021). Species of the genera *Bacteroides* and *Phocaeicola* are ubiquitous commensals, comprising about thirty percent of the human gut microbiota (Salyers 1984). Hence, these microorganisms play an essential role in the colonic ecosystem (Wexler and Goodman 2017). *Phocaeicola vulgatus* (*P. vulgatus*), originally classified as a *Bacteroides* species (García-López et al. 2019), is one of the most numerous organisms within the family *Bacteroidaceae* in the colon (up to 10^10^ per g stool) (Salyers 1984).

To date, members of the phylum Bacteriodetes have not been used for biotechnological processes. However, species belonging to the family *Bacteroidaceae*, including *P. vulgatus*, are known to be highly effective succinate and propionate producers (Flint et al. 2015; Koh et al. 2016; Ríos-Covián et al. 2016; de Vadder and Mithieux 2018) and possess enzyme systems for the degradation of complex polysaccharides (Chassard et al. 2007; Flint et al. 2008; Makki et al. 2018). There is also strong evidence that these organisms are involved in the synthesis of prebiotic and bioactive compounds (Smith and Macfarlane 1996; Russell et al. 2013), which are often associated with human and animal health benefits. Therefore, species of the family *Bacteroidaceae* could be used as platform organisms for the efficient and sustainable conversion of renewable resources, such as xylan and other hemicelluloses (Dodd et al. 2011; Robert et al. 2007), into bioactive compounds and important bulk chemicals. For the production of such substances at biotechnological scales, the accessibility of these organisms for targeted genetic manipulation is desirable. However, few genetic tools are available. Replicative plasmids and integrative transposons have been constructed for increased gene expression (Smith et al. 1992; Wang et al. 2000; Gupta et al. 2003). However, in heterologous gene expression studies, strong *Escherichia coli* (*E. coli*) promoters were shown not to function in *B. fragilis* (Smith et al. 1992). Unlike most other prokaryotes, such as *E. coli*, the unique major sigma factor in *Bacteroidaceae* species binds to a different consensus sequence, separated by a spacer of variable length (generally 19–21 nucleotides) (Bayley et al. 2000; Mastropaolo et al. 2009). Similarly, the ribosome binding site (RBS) differs in comparison to the RBS of *E. coli*. Accordingly, the RBS from species of the family *Bacteroidaceae* has a lower GC content, which results in a lower tendency to form secondary structures (Accetto and Avguštin 2011). These unique promoters and RBS structures make it difficult to transfer genetic systems evolved in bacteria of other phyla (Smith et al. 1992). Furthermore, in genetic and phenotypic studies with intestinal *Bacteroidaceae*, predominantly only two species have been analyzed: *B. fragilis* and *B. thetaiotaomicron* (Salyers et al. 2000; Wexler and Goodman 2017). However, members of the genera *Bacteroides* and *Phocaeicola* exhibit high intra-specific genomic and metabolic diversity (Pasolli et al. 2019). The heterogeneity highlights the need for genetic studies on different species belonging to the family *Bacteroidaceae*.

In this study, we analyzed different tools for the genetic modification of *P. vulgatus*, to enable homologous gene expression and protein production. Besides succinate and propionate, the organism also produces acetate, formate, and lactate. In the central metabolism of *P. vulgatus*, lactate is formed from the reduction of pyruvate. Since lactate is a metabolic end product and is not metabolized further, overproduction of this compound should not have a negative effect on the organism and its metabolic properties. Therefore, homologous expression of the *ldh* gene encoding a D-lactate dehydrogenase was chosen as a proof of concept in order to accomplish the genetic accessibility of *P. vulgatus*. In addition, the overproduction of lactate also provides a link to industrial application as it can be converted into biodegradable polyesters, making it a potential material for environmentally friendly plastics (Chang et al. 1999). Here, we demonstrate that different plasmids can be used as a platform for gene expression in *P. vulgatus*, allowing selective manipulation of the metabolism and thus shifting the ratio of metabolic end products towards the synthesis of lactate.

## Materials and methods

### Materials

All reagents, chemicals, and substrates used in this study were purchased from Carl Roth GmbH (Karlsruhe, Germany) and Sigma-Aldrich (St. Louis, USA). Q5 and One Taq DNA polymerase, restriction endonucleases, T4 ligase, and PCR reagents were bought from New England Biolabs (Ipswich, USA). Oligonucleotides were synthesized by Eurofins Scientific (Ebersberg, Germany).

### Bacterial strains and standard culture conditions

*E. coli* strains were cultivated in lysogeny broth (LB) medium (Miller 1972) at 37 °C, 180 rpm with 300 μg ml^−1^ erythromycin or 100 μg ml^−1^ ampicillin, respectively, for plasmid maintenance. *P. vulgatus* DSM 1447 was obtained from the German Collection of Microorganisms and Cell Cultures (DSMZ; Brunswick, Germany). Cultures were grown anaerobically either in complex brain heart infusion (BHI) medium or in modified Defined Minimal Medium with Glucose (DMMG) in serum flasks sealed with butyl rubber stoppers under an N_2_/CO_2_ atmosphere (80%/20%) (Varel and Bryant 1974). Prior to inoculation, glucose (30 mM), L-cysteine (0.5 g l^−1^) as reducing agent, vitamin K1 (0.1% (v/v)), and hemin (5 mg l^−1^) were added. DMMG was additionally supplemented with 1 ml l^−1^ vitamin solution (Wolin et al. 1963) and potassium butyrate (2 mM). Cells were grown at 37 °C, and growth was quantified by measuring the optical density at 600 nm (OD_600_). Plasmid-containing *P. vulgatus* strains were grown in broth with the addition of erythromycin (100 μg ml^−1^) and gentamycin (200 μg ml^−1^) during selection after conjugation with *E. coli*.

### Molecular cloning

Plasmids used in this study are listed in Table 1. The shuttle vector pG106 was kindly provided by Kevin R. Jones (The Philips Institute for Oral Health Research, Virginia Commonwealth University, Richmond, VA, USA). BLASTp analysis (https://www.ncbi.nlm.nih.gov/) using the biochemically characterized D-LDH from *E. coli* (b1380) was performed resulting in the identification of protein BVU_2499 as a potential D-LDH in *P. vulgatus.* The corresponding gene (*ldh*) was amplified including 250 bp of the upstream region by PCR using Q5 High-fidelity DNA polymerase and primers *ldh*_nativP_*Sal*I_for and *ldh*_*Sph*I_rev (Table 1) with genomic DNA of *P. vulgatus* as a template. For cloning of the fragment in pG106, the PCR products contained the endonuclease restriction sites *Sal*I and *Sph*I at the 5′ and 3′ end, respectively. The fragments were ligated into the corresponding restriction sites of pG106, resulting in the expression vector pG106_*ldh*nP. For insertion of *ldh* in pMM656, the DNA fragment was cloned via the NEBuilder® HiFi DNA Assembly Cloning Kit into the vector following the manufacturer’s protocol. The following primers were used: (1) bb_pMM656_*ldh*nP_fwd and bb_pMM656_*ldh*nP_rev for the amplification of the vector backbone. (2) as_*ldh*nP_pMM656_fwd and as_*ldh*nP_pMM656_rev to amplify the *ldh* gene. The PRhaKIPAO promoter and the downstream encoded gene *nanoluc* of pMM656 were exchanged for the *ldh* gene including 250 bp of the upstream region of the gene. The corresponding plasmid was referred to as pMM656_*ldh*nP. For overproduction of *ldh*, pASK5_*ldh* was constructed. The amplification of *ldh* was performed using primers *ldh*_pASK5_for and *ldh*_pASK5_rev (Table 1), and the fragment was cloned in-frame to the N-terminal Strep-tag into pASK5 vector by restriction with *Bsa*I. Ligated DNA was transformed via heat shock or electroporation in *E. coli* DH5α (pG106) or *E. coli* λpir S17-1 (pMM656). The plasmids were purified from *E. coli* host strains using a plasmid miniprep Kit from New England Biolabs (Ipswich, USA) and were used for transformation in *P. vulgatus*.

**Table 1:** Strains, plasmids, and primers used in this study

**Strains**	**Description**	**Source**
*P. vulgatus* DSM 1447	Wild type	DSMZ^1^
*P. vulgatus* pMM656*_ ldh*nP	ΔtRNA^Ser^ derivate of *P. vulgatus* with chromosomally integrated plasmid pMM656_*ldh*nP	This study
*P. vulgatus* pG106*_ldh*nP	*P. vulgatus* containing plasmid pG106_*ldh*nP	This study
*E. coli* NEB®5-alpha (referred to as *E. coli*)	*fhuA2* Δ(*arg*F-*lac*Z) U169 *pho*A *gln*V44 Φ80 Δ(*lacZ*)M15 *gyr*A96 *rec*A1 *rel*A1 *end*A1 *thi*-1 *hsd*R17	New England Biolabs (Ipswich, USA)
*E. coli* pG106_*ldh*nP	*E. coli* NEB®5-alpha containing plasmid pG106*_ldh*nP	This study
*E. coli* λpir S17-1	sup E44, ΔlacU169 (ΦlacZΔM15), recA1, endA1, hsdR17, thi-1, gyrA96, relA1, λpir phage lysogen	DSMZ^1)^
*E. coli* λpir S17-1 pMM656_*ldh*nP	*E. coli* λpir S17-1 containing plasmid pMM656_*ldh*nP	This study
**Plasmids**	**Description**	**Source**
pASK-IBA.5 (pASK5)	Vector with inducible tetracycline promoter/operator, ampicillin resistance cassette, f1 origin, MCS, and Strep-Tag for N-terminal fusion to a recombinant protein	IBA Göttingen, Germany
pASK5_*ldh*	pASK-IBA5 derivative containing BVU_2499 from *P. vulgatus* DSM 1447	This study
pG106	Vector with erythromycin resistance cassette (*erm*F and *erm*AM), f1 origin, MCS, repB-mob region for *Bacteroides* replication	Jones et al. 2020
pG106_*ldh*nP	pG106 derivative containing BVU_2499 including 250 bp upstream sequence from *P. vulgatus* DSM 1447	This study
pMM656	Vector with erythromycin and ampicillin resistance cassette, NBU2 integrase, luciferase (NanoLuc), R6KOrigin	Mimee et al. 2015
pMM656_*ldh*nP	pMM656 derivative containing BVU_2499 including 250 bp upstream sequence from *P. vulgatus* DSM 1447	This study
**Primer**	**Sequence (5’ ➔ 3’)**	**Restriction site**
bb_pMM656_*ldh*nP_fwd	TGAACTGCACTTGCTTTG	-
bb_pMM656_*ldh*nP_rev	TTTTATGCAAAAAAAGCATGATTTATG	-
as_*ldh*nP_pMM656_fwd	CATGCTTTTTTTGCATAAAATAACGTTTGCAGAATTGTCCG	-
as_*ldh*nP_pMM656_rev	ATCAAAGCAAGTGCAGTTCATTAGACTCTCCCCACCGC	-
*ldh*_nativP_*Sal*I_for	ATTAGTCGACTAACGTTTGCAGAATTGTCC	*Sal*I
*ldh*_*Sph*I_rev	AATAGCATGCTTAGACTCTCCCCACCGCTA	*Sph*I
*ldh*_pASK5_for	ATGGTAGGTCTCAGCGCCGCTTATAAAATAGCTTTTTACGACAC	*Bsa*I
*ldh*_pASK5_rev	ATGGTAGGTCTCATATCAGACTCTCCCCACCGCTACTTC	*Bsa*I
qPCR_LDH_for	TTACGGTGGCGTTGATGTTA	-
qPCR_LDH_rev	TGTTCACGGGCAAAATTGTA	-
qPCR_L23_for	TCGATTCGGCTTTATTGTACG	-
qPCR_L23_rev	CGCCTTCTTTCAATGTTACGA	-
attB2451_for	TGTTCCAGCCATTCATTAATA	-
attB2094_for	ATCTCCTTTATTTAAATAG	-
attB_inErm_rev	ATGGTTTTGCTAAAATGTTA	-

^1^DSMZ, German Collection of Microorganisms, Brunswick, Germany

### Generation of the ldh overexpression mutant *P. vulgatus* pG106_*ldh*nP

Transformation of pG106 via electroporation in *P. vulgates* was performed as described (Smith 1995) in an anaerobic chamber (Coy Laboratory Products, Grass Lakewood MI, USA) under a 49% N_2_/49% CO_2_/2% H_2_ atmosphere. An overnight culture (50 ml) was harvested by centrifugation at 8000 × *g* for 15 min at 4 °C. The pellet was washed twice in 4 ml of cold electroporation buffer (10% glycerol, 1 mM MgCl_2_) and resuspended in 0.5 ml of the same buffer. Fifty microliters of cell suspension and 5 μl plasmid DNA were added to a pre-chilled cuvette (0.2 cm) and incubated on ice for 5 min. The cuvette was placed in an electroporation chamber and pulsed for 6 ms using settings of 2.5 kV and 400 Ω on a Biorad Gene Pulser II (Biorad, Feldkirchen, Germany). Immediately after electroporation, 500 μl of prewarmed BHI medium was added to the cuvette and transferred into 2 ml of prewarmed BHI medium. Regeneration occurred overnight at 37 °C. The next day, 50 μl of each sample was plated on BHI agar plates with erythromycin and incubated anaerobically for 72 h at 37 °C. Transformants were screened by PCR. A positive clone was verified by sequencing and was referred to as *P. vulgatus* pG106_*ldh*nP.

### Genome integration of pMM656_*ldh*nP

pMM656 constructs were conjugated into *P. vulgatus* using *E. coli* S17 λpir, which contains the conjugative machinery of the plasmid RP4 integrated into the chromosome. For mating*,* an overnight culture of *E. coli* S17 λpir was added to *P. vulgatus* at a cell number ratio of 1:5 (v/v). The mating mixture was pelleted, resuspended in a 200 μl BHI medium, spotted onto a filter on a BHI agar plate, and incubated overnight at 37 °C under aerobic conditions. After overnight incubation, cells on the filter were collected in a 3 ml BHI medium, plated on BHI agar plates with gentamycin and erythromycin, and incubated for 48 h at 37 °C under anaerobic conditions. Colony screening by PCR resulted in the identification of clones containing pMM656 or pMM656_ldhnP inserted in the correct chromosomal location at the tRNA^Ser^ gene (BVU_2094). The mutants were referred to as *P. vulgatus* pMM656 and *P. vulgatus* pMM656_*ldh*nP and were verified by PCR and sequencing (Fig. S1). For this purpose, the primer pair attB2451_for or attB2094_for and attB_inErm_rev was used, which annealed at the plasmid backbone as well as at BVU_2451 or BVU_2094 located on the chromosome.

### Overexpression and purification of *ldh*

For protein production, overnight cultures of *E. coli* DH5α (5 ml) harbouring plasmid pASK5_*ldh* were used to inoculate 500 ml LB medium and were incubated at 37 °C and 180 rpm in shaker flasks. Cells were grown to an OD_600_ of 0.4 and induced by adding 0.2 μg ml^−1^ anhydrotetracycline to start production of D-LDH (16 h, 16 °C, 180 rpm). Cells were harvested by centrifugation at 8000 × *g* and 4 °C for 20 min and resuspended in 5 ml buffer W, containing 100 mM Tris-HCl, 150 mM NaCl, and pH 8. Cells were lysed by sonification and cell debris were separated by centrifugation at 13,000 × *g* and 4 °C for 10 min. For purification of D-LDH, the culture supernatant was applied to a gravity flow Strep-Tactin Superflow affinity column (IBA GmbH, Göttingen, Germany). Buffer W supplemented with 2.5 mM D-desthiobiotin was used as eluent. The concentration of purified D-LDH was determined by a Bradford assay (Bradford 1976). For protein visualization, polyacrylamide gel electrophoresis was performed (Laemmli 1970) and protein bands were detected via silver staining as described (Blum et al. 1987). Immunoblotting was done according to Towbin et al. (1979) and the protein was detected colorimetrically by the fused Strep-Tag using the Strep-Tactin® HRP Conjugate (IBA GmbH, Göttingen, Germany).

### Luciferase assay

Two hundred microliters of cultures of *P. vulgatus* pMM656 were incubated in 96-well plates with different concentrations of rhamnose for induction of the PRhaKIPOA promoter overnight at 37 °C in an Infinite 200 PRO NanoQuant Microplate Reader (Tecan, Männedorf, Switzerland) in an anaerobic chamber filled with N_2_/CO_2_/H_2_ (49%/49%/2%). The cultures were lysed by adding 20 μl of PopCulture reagent (EMD Millipore, Darmstadt, Germany) supplemented with lysozyme solution (160 U). The NanoLuc® luciferase assay (Promega, Walldorf, Germany) was performed according to the manufacturer’s instructions using 10 μl *P. vulgatus* cell lysate and 10 μl NanoLuc reaction buffer including the substrate furimazine. NanoLuc oxidizes furimazine and produces bioluminescence (*E*_max_ = 460 nm) with a signal half-life of 2 h. Luciferase activity was measured with an integration time of 1 s in an Infinite 200 PRO NanoQuant Microplate Reader (Tecan, Männedorf, Switzerland) under aerobic conditions. The corresponding relative light units (RLU) were normalized to OD1.

### Determination of the ldh copy number

The relative number of *ldh* genes in *P. vulgatus* pG106_*ldh*nP, *P. vulgatus* pMM656_*ldh*nP, and the wild type was determined by qPCR. The gene *l23* (BVU_0803), encoding the ribosomal protein L23, is present once on the chromosome of *P. vulgatus* and was used as a reference. Gene-specific primers (qPCR_LDH_for, qPCR_LDH_rev, qPCR_L23_for, and qPCR_L23_rev; Table 1) for qPCR experiments were designed using the primer3 software (https://bioinfo.ut.ee/primer3/) (Koressaar and Remm 2007; Untergasser et al. 2012; Koressaar et al. 2018). All primers had an almost identical annealing temperature, and their amplicons had a similar size (180–200 bp), a similar melting temperature (58–60°C), and a similar GC % content. Strains were grown overnight in the BHI medium. After 24 h, each culture was diluted to OD_600_ = 0.5, and 1 ml was centrifuged and washed in a phosphate-buffered saline solution (8.0 g NaCl, 0.2 g KCl, 1.4 g Na_2_HPO_4_, 0.27 g KH_2_PO_4_ per 1 l H_2_O). Cell pellets were resuspended in 1 ml nuclease-free water and boiled for 10 min to lyse the cells. The cell lysates were diluted and supplemented with the Luna® Universal qPCR Master Mix (New England Biolabs, Ipswich, USA). qPCR was performed on a CFX Connect Real-Timer PCR Detection System (BioRad, Munich, Germany). For calculations of ΔCt values, the quantification cycle (Ct) of the gene of interest was subtracted from the Ct value of the reference gene (*l23*). Subsequently, ΔΔCt values and the corresponding fold change were calculated in relation to the wild type. The fold change was calculated using the formula 2^−ΔΔCt^.

### RT-qPCR analysis of gene expression

The analysis of transcript abundance of the *ldh* gene of *P. vulgatus* strains was performed by RT-qPCR experiments. Total RNA from *P. vulgatus* was isolated from 50 ml cultures grown in the BHI medium to mid-exponential phase. The total RNA Miniprep Kit (New England Biolabs, Ipswich, USA) was used to purify RNA from cells following the manufacturer’s protocol. RNA samples were treated with DNase I to remove residual DNA, and RNA concentrations were measured spectrophotometrically using a BioSpectrometer® (Eppendorf, Hamburg, Germany). Additionally, control PCR experiments were performed to confirm that RNA samples did not contain DNA contaminations. Gene-specific primers used for RT-qPCR experiments were the same as for qPCR experiments. The gene *l23*, encoding the ribosomal protein L23, was chosen as a reference. RT-qPCR reactions were performed with a Luna® Universal One-Step RT-qPCR Kit (New England Biolabs, Ipswich, USA). Each PCR reaction contained 200 ng of purified RNA. For temperature cycling and fluorescence measurement, a cycler CFX Connect™ and a suitable software (BioRad, Munich, Germany) were used. Melting curve analysis resulted in single peaks for the respective PCR fragments, confirming specific products from PCR reactions. To determine transcript abundance, the ΔΔCt values and the fold change were evaluated as described above.

### Preparation of cell-free extract and enzyme assay

Cell-free extract was prepared from *P. vulgatus* cells grown in a 50 ml BHI medium. Cells were harvested in the exponential growth phase by centrifugation (10,000 × *g*, 15 min, 4°C) and resuspended in 10 ml buffer W, containing 100 mM Tris-HCl, 150 mM NaCl, and pH 8. Cells were lysed by sonification and cellular debris were removed by centrifugation for 15 min at 8000 × *g* and 4 °C. Protein concentrations of the cell-free extracts were determined as described (Bradford 1976). The 1 ml assay contained 100 mM potassium phosphate buffer pH 7, 2.5 μM NADH, 5 mM pyruvate, and different concentrations of cell-free extract. The activity was measured photometrically at 340 nm. The temperature optimum of purified D-LDH was determined at pH 7 and temperatures ranging from 30 to 80 °C. The optimum pH of D-LDH was determined using a combined buffer system containing sodium acetate, Tris-HCl, potassium dihydrogen phosphate, and dipotassium hydrogen phosphate (50 mM each) in the range from pH 4 to 9 at 37 °C. Enzyme kinetics were measured by varying substrate concentration (0–50 mM) at physiological pH (7) and temperature (37 °C).

### HPLC analysis of culture supernatants

The analysis of substrate and product concentrations was performed by HPLC (Knauer Smartline HPLC system, Knauer GmbH, Berlin, Germany) with an Aminex HPX-87H column (BioRad, Munich, Germany, 300 mm × 7.8 mm) using 5 mM H_2_SO_4_ as mobile phase. One milliliter of culture was harvested by centrifugation (10,000 × *g*, 2 min and 10 °C) at different OD_600_ in the exponential growth phase. Metabolic end products and the substrate glucose were separated at a column temperature of 65 °C with a flow rate of 0.6 ml min^−1^ and detected by a refractive index detector. Concentrations were calculated by comparison to corresponding calibration curves.

## Results

### Genetic tools for homologous gene expression in *P. vulgatus*

For homologous expression of genes, the shuttle vector pG106 (Jones et al. 2020) and the genome-integrative vector pMM656 (Mimee et al. 2015) were used. Plasmid pG106 comprises a 5 kb Mob-Rep region, which contains an oriV for the initiation of plasmid replication in *P. vulgatus*. For the selection of *E. coli* and *P. vulgatus*, the vector encodes an *erm*F-*erm*AM cassette. Stable transformation of pG106 into *P. vulgatus* was achieved by electroporation.

The pMM656 plasmid encodes an *intN2* tyrosine integrase, which mediates sequence-specific recombination between the attN2 site of pMM656 and one of two attPV sites located in the 3′ ends of the two tRNA^Ser^ genes, BVU_2451 and BVU_2094, on the *P. vulgatus* chromosome (Mimee et al. 2015). Insertion of the pMM656 plasmid inactivates one tRNA^Ser^ gene, so simultaneous insertion in BVU_2451 and BVU_2094 is unlikely due to the essentiality of tRNA^Ser^. The gene encoding the luciferase NanoLuc functions as a reporter gene. Transformation of pMM656 into *P. vulgatus* was performed by biparental conjugation.

**Fig. 1:**
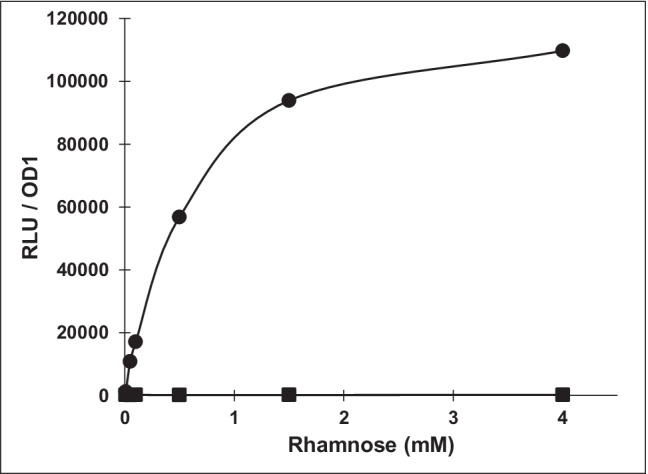
NanoLuc activity under the control of the rhamnose inducible promoter. *P. vulgatus* pMM656 cells were incubated for 24 h at 37 °C without rhamnose or serial dilutions of rhamnose starting at 0.05 mM. After lysis, the cell lysate was treated as described in “Materials and methods”. Relative light unit (RLU) values were normalized to OD 1. The experiment was conducted four times and the respective standard deviations are indicated by error bars

To evaluate the functionality of pMM656 in *P. vulgatus*, the luciferase reporter system encoded on the integrated vector was used. Expression of the *nanoluc* gene was mediated by the upstream promoter-RBS region (PRhaKIPAO) of the rhamnulose kinase gene (BT_3763) of *B. thetaiotaomicron* (Mimee et al. 2015). Gene expression was dependent on the concentration of rhamnose (0–4 mM) and demonstrated a response curve with an output dynamic range of 525-fold. Without induction by rhamnose, transcription of *nanoluc* could not be detected (Fig. 1). Above a rhamnose concentration of 4 mM maximal expression was observed, which did not influence the growth of *P. vulgatus* (data not shown). In this context, it is to mention that the functionality of the *nanoluc* gene was already shown for several *Bacteroides* strains and *P. vulgatus* by using anhydrotetracycline-inducible *tet* promoter constructs (Lim et al. 2017).

### Application of expression vector pG106 and integration vector pMM656 for the redirection of the carbon flow in *P. vulgatus*

The metabolic versatile *P. vulgatus* produces acetate, formate, and lactate by fermentation and succinate by fumarate respiration. Furthermore, succinate can be decarboxylated to propionate. Substrates are complex heteropolysaccharides, which are degraded to pentoses or hexoses. When growing on a DMMG medium with glucose as substrate, the lactate production is very low compared to the other fermentative end products.

**Fig. 2:**
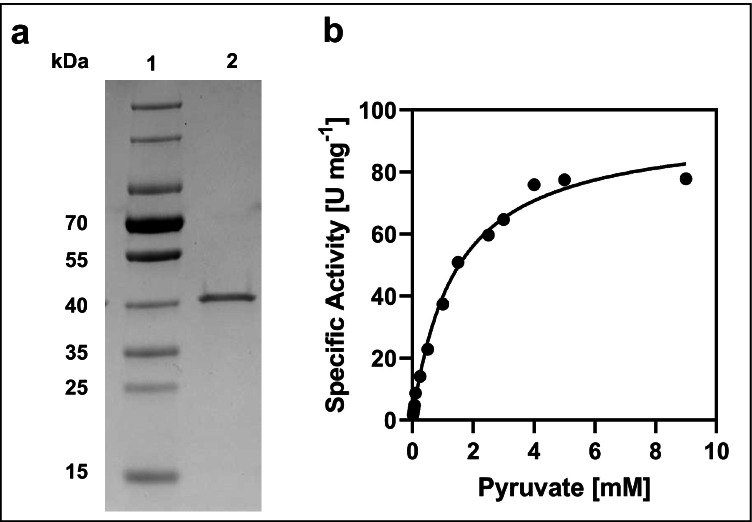
Characterization of purified D-LDH from *P. vulgatus*, overproduced in *E. coli*. **a** SDS/PAGE analysis of recombinant D-LDH using silver staining. Lane 1: PageRuler^TM^ Prestained Protein Ladder (ThermoFisher Scientific). Lane 2: purified D-LDH (1.5 μg protein). **b** Michaelis–Menten kinetics. For the detection of NADH oxidation, purified D-LDH and pyruvate in different concentrations were added and the change in absorbance at 340 nm was analyzed at 37 °C. The enzyme assay (1 ml) contained 100 mM K-phosphate buffer, pH 7, and 250 μM NADH. The pyruvate concentration was varied between 0.02 and 9 mM. Nonlinear regression of the Michaelis–Menten data was used to calculate the kinetic constants *V*_max_ and *K*_m_ with the program GraphPad Prism

The question arose whether it is possible to increase the concentration of lactate by overexpressing a lactate dehydrogenase and thus modifying the central metabolism of *P. vulgatus*. To answer this question, the genome of *P. vulgatus* was screened for the presence of a gene encoding a lactate dehydrogenase. Based on bioinformatic analyses, we identified a *ldh* gene (BVU_2499) that potentially codes for a D-lactate dehydrogenase (D-LDH, EC 1.1.1.28). In general, NAD-dependent D-LDH catalyse the reduction of pyruvate to D-lactate coupled to the oxidation of NADH to NAD^+^ (Holbrook et al 1975). Compared to L-LDHs (EC 1.1.1.27), which are widely distributed in vertebrates and higher plants (Holbrook et al. 1975; Garvie 1980), D-LDHs are found only in some invertebrates and mainly in microorganisms (Garvie 1980). The corresponding motifs that characterize D-LDHs (Furukawa et al. 2018) can be found in the D-LDH amino acid sequence of *P. vulgatus* (Fig. S2). To verify the function of BVU_2499, the corresponding gene was amplified, ligated into the expression vector pASK-IBA5, and overproduced in *E. coli*. The N-terminal fused Strep-tag sequence allowed purification via Strep-Tactin affinity chromatography, leading to a total yield of 27 mg recombinant protein per l culture. The predicted molecular mass of the recombinant tagged protein was 39.1 kDa. Separation by polyacrylamide gel electrophoresis and subsequent silver staining revealed a single band within the elution fraction that corresponded to the expected size (Fig. 2a).

To analyze the activity of the enzyme, the oxidation of NADH to NAD^+^ during the conversion of pyruvate to D-lactate was measured at a wavelength of 340 nm. Purified D-LDH showed the highest specific activity at 60 °C and pH 7. However, the half-life of the enzyme was less than 2 min at this temperature, indicating that the enzyme was not thermostable. The optimal growth temperature of *P. vulgatus* is 37 °C, which correlates with the physiological temperature of the human gut, the natural habitat of *P. vulgatus*. At this temperature, the enzyme was stable over several hours and showed 86.6% of the activity at 60 °C (data not shown). Kinetic parameters were determined from the nonlinear regression of the Michaelis–Menten data (Fig. 2b). Using pyruvate as a substrate, the *K*_M_ and *V*_max_ values were 1.4 ± 0.1 mM and 95.6 ± 3.0 U/ mg protein at a temperature of 37 °C, respectively. These results correspond to values given in the literature (Stoll et al. 1998; Furukawa et al. 2014; Kochhar et al. 1992).

**Fig. 3:**
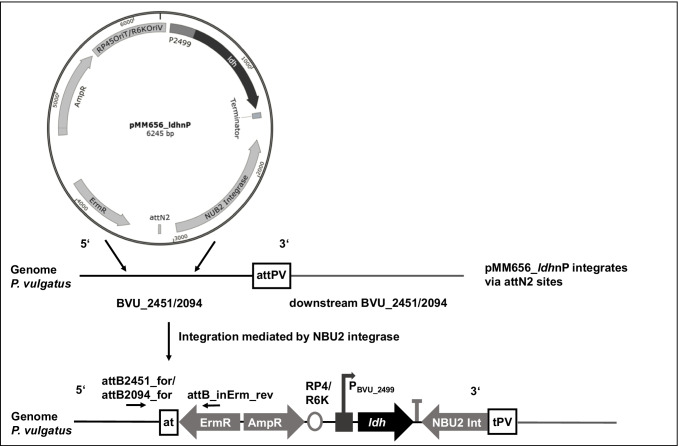
Schematic representation of the integration of pMM656_*ldh*nP into the genome of *P. vulgatus*. The promoter PRhaKIPAO and the coding gene of NanoLuc of pMM656 were replaced with the *ldh* gene (BVU_2499) of *P. vulgatus* including 250 bp of the upstream region. The tyrosine integrase NBU2 Int catalyzed the stable integration of pMM656_*ldh*nP into one of two attN2 sites in the *P. vulgatus* genome. The two attN2 sites are located at the 3′ ends of tRNA^Ser^ genes (BVU_2451 at nt 3,152,564 and BVU_2094 at nt 2,710,348, respectively). AmpR, ampicillin resistance cassette; ErmR, erythromycin resistance cassette; RP4, origin of transfer; R6K, origin of replication. Black horizontal arrows indicate the position of the primer pair attB2451_for/attB2094_for and attB_inErm_rev. The primers were used to generate a DNA fragment for sequencing and to determine the position of integration

For homologous expression of *ldh* from *P. vulgatus*, the gene was cloned into the multiple cloning site of the shuttle vector pG106, resulting in the plasmid pG106_*ldh*nP. Since little is known about promoter structures in *P. vulgatus*, 250 bp of the upstream region of the gene was also cloned for initiation of transcription. For homologous expression by the genome-integrative vector pMM656, the *B. thetaiotaomicron*-derived promoter PRhaKIPAO and the coding gene for NanoLuc from pMM656 were substituted against *ldh* (BVU_2499) including the 250 bp upstream region with the presumed promoter region (Fig. 3). The corresponding plasmid was referred to as pMM656_*ldh*nP. After conjugation, the DNA sequence of the PCR fragment generated by the primer pair attB2094_for and attB_inErm_rev demonstrated that the vector was integrated into BVU_2094 on the genome of *P. vulgatus*, mediated via the attN2 site of pMM656 (Fig. S1).

**Fig. 4:**
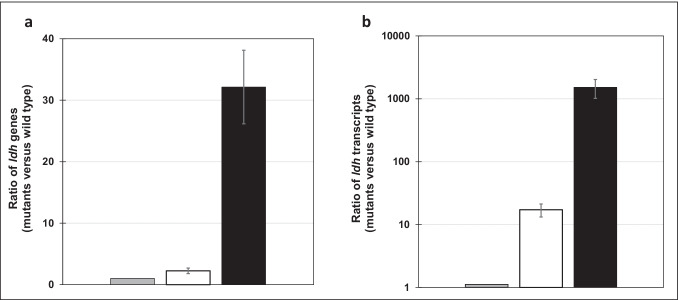
*ldh* copy number (a) and *ldh* transcripts abundance (b) in the wild type and genetically modified mutants of *P. vulgatus*. **a** To estimate the copy number, qPCR was performed to compare chromosomal and plasmid-encoded *ldh* of the mutant strains with the single-copy chromosomal *ldh* of the wild type strain. **b** The relative amount of *ldh* transcripts was analyzed by RT-qPCR. Experiments shown in **a** and **b** were conducted in duplicate using DNA or RNA preparation from three different cultures harvested in the mid-exponential growth phase. ΔCt values were determined by subtracting the average Ct values of the *ldh* gene (BVU_2499) from the reference gene encoding the ribosomal protein L23 (BVU_0803). Ratios were calculated from ΔΔCt values using the function 2^–ΔΔCt^. Standard deviations are indicated by error bars. Gray bars, wild type; white bars*, P. vulgatus* pMM656_ldhnP; black bars, *P. vulgatus* pG106_ldhnP

To analyze the conditions for homologous overproduction of D-LDH in *P. vulgatus*, the copy number of the *ldh* gene in each construct was determined by qPCR. The quantification cycle (Ct) of the gene encoding the ribosomal protein L23 (BVU_0803) was used as a reference. It should be noted that the *ldh* gene and the *l23* gene are encoded only once in the genome of the wild type. As expected, the highest amount of amplicons of the *ldh* gene was detected in *P. vulgatus* pG106_*ldh*nP containing the plasmid-encoded version of the *ldh* gene (Fig. 4a). The number of PCR fragments from the *ldh* gene was 32 ± 6.0-fold higher in *P. vulgatus* pG106_*ldh*nP and 2.2 ± 0.4-fold higher in *P. vulgatus* pMM656_*ldh*nP, respectively compared to the wild type containing a single *ldh* copy. Hence, it is evident that besides the chromosomal version of the *ldh* gene, a second copy was present due to the integration of the vector pMM656_*ldh*nP into the genome of *P. vulgatus* pMM656_*ldh*nP via one of the two attN2 sites. It can also be concluded that 32 ± 6.0 copies of the *ldh* gene were present in the plasmid-containing strain, indicating that the copy number of the shuttle vector pG106_*ldh*nP is in the range of 30.

To determine the transcript abundance, RT-qPCR was performed to compare the expression level of chromosomal and plasmid-encoded *ldh* of the mutant strains with the expression level of the single-copy chromosomal *ldh* gene of the wild-type strain. Total RNA was extracted from each *P. vulgatus* mutant and the wild type grown in BHI and harvested in the mid-log phase. mRNA concentrations were quantified using the same gene-specific primers used in qPCR experiments (Table 1) to determine the copy number of the *ldh* gene. Compared to the wild type, a 1521 ± 506-fold higher transcript abundance was detected in *P. vulgatus* carrying the shuttle vector pG106_*ldh*nP. In *P. vulgatus* harbouring the genome-integrative vector pMM656_*ldh*nP, the transcript level of the *ldh* gene was 17 ± 4-fold higher in comparison to the wild-type strain (Fig. 4b). These data indicate that *P. vulgatus* expressed *ldh* in considerably higher amounts in the shuttle vector containing strain, and is a suitable system for the overproduction of LDH as a model for the modulation of the intracellular carbon flow.

### Enzyme activity of LDH in cell-free extract of *P. vulgatus*

**Fig. 5:**
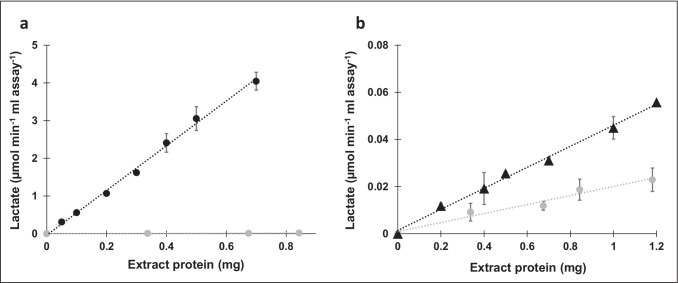
Photometric analysis of enzyme activity of D-LDH in cell-free extract. Determination of the activity of D-LDH from *P. vulgatus* pG106_*ldh*nP (**a**) and *P. vulgatus* pMM656_*ldh*nP (**b**) compared to wild type. Enzyme activities were assayed using cell-free extract by measuring the change in absorbance at 340 nm corresponding to the oxidation of NADH to NAD^+^ during the conversion of pyruvate to D-lactate. The experiment was performed in duplicate using the cell-free extract from two different cultures and the respective standard deviations are indicated by error bars. Black circles, *P. vulgatus* pG106_*ldh*nP; black triangles, *P. vulgatus* pMM656_*ldh*nP; gray circles, wild type

To evaluate the effect of the *ldh* transcription levels on the corresponding enzyme yields, D-LDH activity assays were performed using cytoplasmic fractions of the investigated *P. vulgatus* strains. The change of absorbance at a wavelength of 340 nm was measured, corresponding to the oxidation of NADH to NAD^+^ during the conversion of pyruvate to D-lactate. A much higher D-LDH activity was detected in the cytoplasm of *P. vulgatus* pG106_*ldh*nP (5.9 ± 0.4 U/mg protein) compared to *P. vulgatus* pMM656_*ldh*nP (0.046 ± 0.002 U/mg protein) and the wild-type strain of *P. vulgatus* (0.025 ± 0.002 U/mg protein) (Fig. 5). All assays revealed a stringent correlation between the amount of D-LDH and the lactate formation rate. Considering the ratios of enzyme activities of the D-LDH normalized to that of the wild type, the activity of the D-LDH overexpressing mutant *P. vulgatus* pG106_*ldh*nP and the genome-integrative mutant *P. vulgatus* pMM656_*ldh*nP was 236 and 1.8 times higher, respectively.

### Influence of LDH activity on the ratio of metabolic end products of *P. vulgatus*

To test the effect of the increased abundance of *ldh* transcripts and the elevated activity of D-LDH on the central metabolism of *P. vulgatus* pG106_*ldh*nP and *P. vulgatus* pMM656_*ldh*nP in comparison to the wild type, the end products of glucose conversion were determined by HPLC analysis. Several cultures of the respective strains were grown in a minimal medium with glucose as the substrate. Cells were harvested at different optical densities in the exponential growth phase, and the supernatant was used for product analysis. For the exact determination of the growth behaviour, product and substrate concentrations were correlated with the dry weight (DW) of the respective culture.

**Fig. 6:**
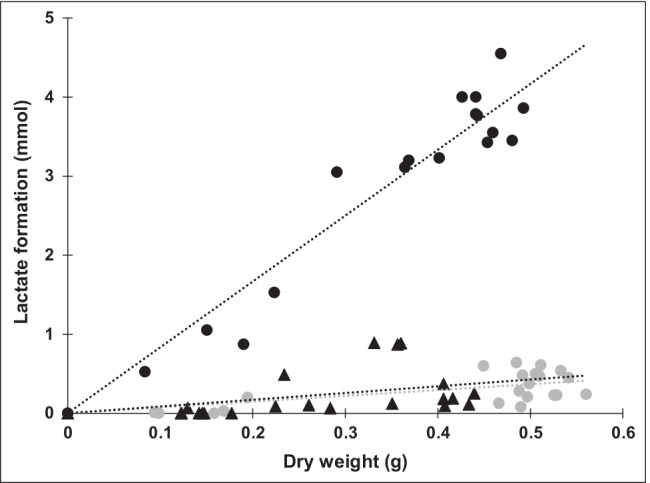
Lactate formation by the wild type and the mutants. *P. vulgatus* pG106_*ldh*nP (black circles), *P. vulgatus* pMM656_*ldh*nP (black triangles), and the wild type (gray circles) were grown in minimal medium with glucose as substrate. At least 16 cultures of each strain were harvested in the exponential growth phase and the supernatants were analyzed by HPLC. The lactate concentration was correlated to the dry weight of the corresponding culture. For a culture with an optical density of 1.0, the DW was 360 mg per liter culture. Lactate yields per g DW were calculated from the slope of the regression lines

**Fig. 7:**
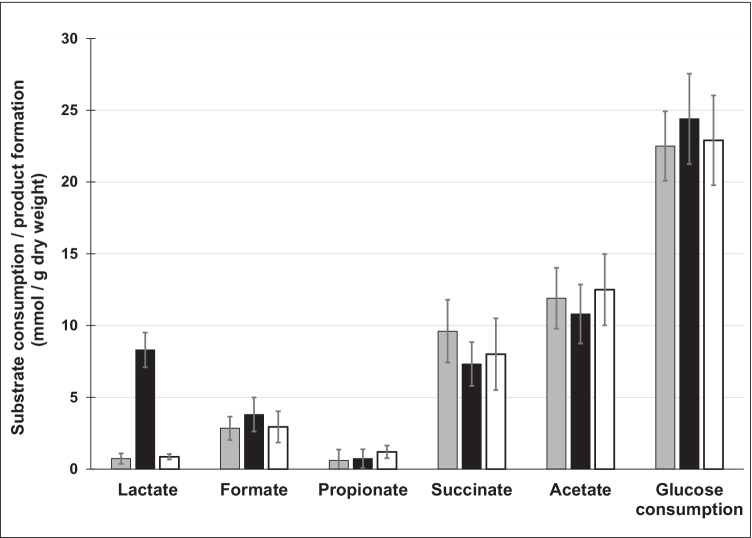
Substrate consumption and product formation. *P. vulgatus* pG106_l*dh*nP (black), *P. vulgatus* pMM656_*ldh*nP (white), and the wild type (gray) were grown in DMMG medium. Cultures were harvested and the supernatants analyzed by HPLC. The concentrations of the metabolic end products were correlated to the dry weight of the corresponding cultures. The values represent the average of at least 16 different cultures for each strain (cultures from Fig. 6). Standard deviations are indicated by error bars

As expected, succinate, acetate, lactate, formate, and propionate were found as products of glucose fermentation. However, a major difference was observed with respect to the formation of lactate (Fig. 6). The wild-type strain of *P. vulgatus* produced 0.73 ± 0.36 mmol lactate/g DW. With 0.85 ± 0.2 mmol/g DW, *P. vulgatus* pMM656_*ldh*nP formed a little more lactate than the wild-type strain. In the shuttle vector carrying mutant *P. vulgatus* pG106_*ldh*nP, a level of 8.3 ± 1.2 mmol/g DW lactate was detected. Compared to the wild type, this corresponded to a ratio of 1:11.4, and compared to *P. vulgatus* pMM656_*ldh*nP, 9.8 times as much lactate was produced (Fig. 6). Substrate consumption of the individual strains was also different (Fig. 7). The *P. vulgatus* wild type and the mutant containing a second chromosomal copy of the *ldh* gene consumed 22.5 ± 2.4 and 22.9 ± 3.13 mmol glucose/g DW, respectively, whereas the plasmid-harbouring strain metabolized significantly (*p* ≤ 0.05) more glucose (24.4 ± 3.15 mmol glucose/g DW) compared to the wild type (Figs. 7, S3). A closer look at the products indicated that the formation of succinate and acetate was slightly lower in *P. vulgatus* pG106_*ldh*nP compared to the wild type and *P. vulgatus* pMM656_*ldh*nP. The concentrations of formate and propionate were in the same range of 1–2 mmol/g DW.

The additional carbon required for the increased amount of lactate was reflected in a slightly increased glucose consumption and a decreased amount of succinate and acetate produced by *P. vulgatus* pG106_*ldh*nP. In summary, the data clearly indicate that the plasmid-based overexpression of the *ldh* gene resulted in a highly increased number of transcripts and enhanced activity of the D-LDH within the cells of *P. vulgatus* pG106_*ldh*nP. This in turn leads to a shift in the product spectrum towards the increased production of lactate.

## Discussion

The human gastrointestinal tract represents one of the densest microbial communities known in nature. This habitat is dominated mainly by the two bacterial phyla Firmicutes and Bacteroidetes (Faith et al. 2013; Human Microbiome Project Consortium 2012). The latter phylum comprises genera such as *Bacteroides, Alistipes, Parabacteroides*, *Phocaeicola,* and *Prevotella* (Wexler and Goodman 2017). Recently, some members of the genus *Bacteroides* were taxonomically separated and assigned to the genus *Phocaeicola*. Among these species is *P. vulgatus* as one of the most prominent gut bacteria (García-López et al. 2019).

Members of the mentioned genera possess enzyme systems for the degradation of complex polysaccharides (Hill 1995; Ley et al. 2008; Flint et al. 2012), which cannot be digested by the human host. Fermentation of these dietary fibres ultimately leads to the formation of short-chain fatty acids (mainly butyrate, propionate, and acetate), which are released into the intestinal lumen and taken up by host colonocytes (Fischbach and Sonnenburg 2011; Tremaroli and Bäckhed 2012; den Besten et al. 2013). This production of organic acids from biomass conversion is gaining economic attention (McKinlay et al. 2007; Baumann and Westermann 2016). Since *Bacteroides* spp. and *Phocaeicola* spp. are known to be highly effective producers of succinate, acetate, and propionate (Scheifinger and Wolin 1973; Salonen et al. 2014), the production of these organic acids by the corresponding organisms could be of great interest in future biotechnological applications.

In general, the production of these platform chemicals on a biotechnological scale by members of these genera requires genetic manipulation of the metabolism towards the improvement of the corresponding biochemical pathways. There is already a variety of genetic tools available for members of the family *Bacteriodaceae*, which includes the genera *Bacteroides* and *Phocaeicola*. However, most of the genetic tools were designed for *B. thetaiotaomicron*, *B. fragilis,* and *B. ovatus*, all classified as risk group 2 organisms and therefore not applicable for biotechnological applications. Moreover, it was shown that *Bacteroides* species possess unique promoter structures (Bayley et al. 2000; Vingadassalom et al. 2005) and ribosome binding sites (Smith et al. 1992; Accetto and Avguštin 2011; Wegmann et al. 2013). Hence, genetic systems for targeted gene manipulation developed for members of the Proteobacteria, especially *E. coli*, cannot be used in members of the family *Bacteriodaceae*. Many *Bacteroides* spp*.* possess small cryptic plasmids associated with transmissible antibiotic resistance and genes for conjugal transfer (Smith 1995), which can function as a basis for the construction of conjugative shuttle vectors. Some shuttle vectors, with constitutive or inducible promoters, have already been employed for regulated gene expression in *B. thetaiotaomicron, B. fragilis,* and *B. ovatus* (Smith 1995; Hamady et al. 2008; Parker and Smith 2012; Horn et al. 2016; Lim et al. 2017; Jones et al. 2020), but most are not commercially available. In addition, a group of plasmids was identified that enable transposon mutagenesis based on a marine transposon (Goodman et al. 2009). Deletion mutants in *Bacteroides* spp. were generated by standard allelic replacement techniques using conjugal suicide vectors in combination with erythromycin selection (Chatzidaki-Livanis et al. 2009; Nakayama-Imaohji et al. 2012) or antibacterial counterselection markers (Bencivenga-Barry et al. 2020).

Here, we used the commercially available integration vector pMM656 (Mimee et al. 2015) and the shuttle vector pG106 (Jones et al. 2020) for homologous gene expression in *P. vulgatus*. The function of pMM656 is based on the integration of the vectors into the so-called “attN sites”, which are located at the 3’ end of the genes of the tRNAs for serine. Bioinformatic analysis revealed that these attN sites are also present in *P. vulgatus*. Our results showed that the *B. thetaiotaomicron*–specific and rhamnose-induced promoter upstream of the NanoLuc reporter gene worked as expected, indicated by the rhamnose-dependent expression of the luciferase. Furthermore, the exchange of this promoter/gene sequence against the *ldh* gene led to an increase of the corresponding mRNA by 17-fold. Hence, pMM656 is a suitable tool for applications of molecular cloning including complementation and gene expression studies in *P. vulgatus*.

The second plasmid pG106 applied in this study is used as a shuttle vector for genetic manipulation in *B. thetaiotaomicron* or *Porphyromonas* spp. (Jones et al. 2020). A copy number of about six was determined for *B. thetaiotaomicron* whereas we detected about 30 copies indicating that the plasmid has a medium copy number in *P. vulgatus*. To test the genetic accessibility of *P. vulgatus*, homologous overexpression of the *ldh* gene was chosen as a proof of concept in this study. Therefore, the *ldh* gene and its upstream region (250 bp) were cloned into pG106 replacing the NanoLuc reporter gene and its promoter. The plasmid was referred to as pG106_*ldh*nP. In relation to the copy number of the *ldh* gene, an approx. 1500-fold increase in the *ldh* transcript concentration was observed in *P. vulgatus* pG106_*ldh*nP in comparison to the parental strain. Hence, the results also indicate that the 5’ UTR of the *ldh* gene encodes a strong promoter. Representatives of the Bacteroidetes were shown to typically have promoter structures with a TAnn(T)TTG and (T)TTG region located 7 base pairs and 33 base pairs upstream of the transcription start of the corresponding gene, respectively (Chen et al. 2007; Lim et al. 2017; Bayley et al. 2000). Corresponding motifs are highly conserved in the 5’ UTR region of the *ldh* gene of *P. vulgatus*. In addition to the enhanced transcript concentration, the enzyme activity of D-LDH increased by a factor of more than 200 in the overexpression strain, compared to the wild type. This result suggests that this tool is a suitable system for the overproduction of proteins in *P. vulgatus*.

The increased amount of D-LDH in the cells had a severe impact on the end product composition. Under the growth condition employed, *P. vulgatus* produced only minor amounts of lactate (< 1 mmol/g DW). The enhanced activity of D-LDH in *P. vulgatus* pG106_*ldh*nP led to an 11.4 times higher production rate of lactate compared to the parental strain. The final concentration was in the range of 8.3 mmol/g DW, which was comparable to the concentration of the major end products succinate and acetate. Considering the amount of glucose used for the catabolic metabolism (14.4 mmol glucose/g DW), the proportion of lactate in the overall catabolic carbon flow increased from 2.9% (wild type) to 28.8% in the LDH-overproducing mutant. The increased amount of lactate formed by *P. vulgatus* pG106_*ldh*nP compared to the wild-type strain was probably compensated by the higher consumption of glucose (plus 1.9 mmol/g DW) and the lower amount of the metabolic end products succinate (minus 2.3 mmol/g DW) and acetate (minus 1.1 mmol/g DW).

To date, strains of the family *Bacteroidaceae* have not been used for biotechnological processes. However, it is known that members of this taxonomical group are highly effective succinate and propionate producers and possess sophisticated enzyme systems for the degradation of complex polysaccharides. In the future, these species could function as platform organisms for the efficient and sustainable conversion of renewable resources into bioactive compounds and important bulk chemicals. However, a genetic system would be desirable to allow a defined modelling of the intracellular carbon flux. Here, we demonstrated that *P. vulgatus* was genetically accessible and that appropriate expression systems were applicable. Thus, this approach formed the basis for targeted genetic optimization of *P. vulgatus*, namely the homologous expression of the *ldh* gene as a proof of concept for the manipulation of the central carbon flow. Furthermore, improvement of lactate production could be possible by the deletion of genes encoding enzymes, which are involved in the synthesis of other metabolic end products. Examples are the phosphoenolpyruvate carboxykinase or fumarate reductase to prevent the formation of succinate and propionate as well as the pyruvate: formate lyase to stop the synthesis of formate (Franke and Deppenmeier 2018). In the long run, the results could also strengthen the research on the gut microbiota in relation to medically relevant *Bacteroides* and *Phocaeicola* spp..

## Supplementary Information

Below is the link to the electronic supplementary material.

Supplementary file1 (PDF 232 KB)

## Data Availability

Data are available upon request from the authors.
