# Supplementary material for: Genetic tools for the redirection of the central carbon flow towards the production of lactate in the human gut bacterium Phocaeicola (Bacteroides) vulgatus
Source: Appl Microbiol Biotechnol. 2022 Jan 26;106(3):1211–25. doi: 10.1007/s00253-022-11777-6 (PMC8816746; doi:10.1007/s00253-022-11777-6)
Supplement: Supplementary file 1 — Supplementary file1 (PDF 232 KB) [file 253_2022_11777_MOESM1_ESM.pdf]

Supplementary Material for:

Applied Microbiology & Biotechnology

Genetic tools for the redirection of the central carbon flow towards the production of lactate in the human gut bacterium *Phocaeicola (Bacteroides) vulgatus*

Rebecca Lück and Uwe Deppenmeier*

Institute of Microbiology and Biotechnology, University of Bonn, Meckenheimer Allee 168, 53115 Bonn, Germany

*For correspondence. E-mail: udeppen@uni-bonn.de; Tel. +49-228-735590; Fax +49-228-737576.

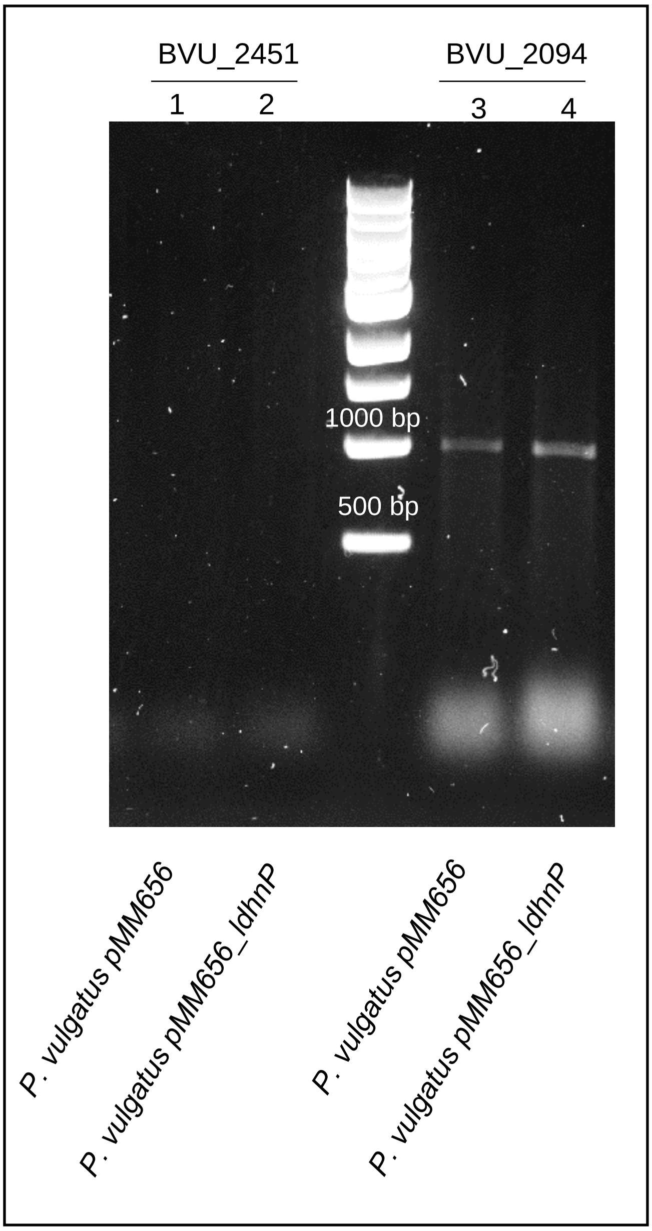

Fig. S1 PCR verification of the genomic integration of pMM656 and pMM656_*ldh*nP into the gene BVU_2094 of *P. vulgatus*. Vector integration can occur between the attN2 site of pMM656 and one of two attPV sites at the 3′ ends of the two tRNA^{Ser} genes, BVU_2451 and BVU_2094, on the *P. vulgatus* chromosome. Primer attB_inErm_rev and attB2451_for or attB2094_for were used to detect integration, with attB_inErm_rev binding in the plasmid backbone of pMM656 and attB2451_for/attB2094_for binding in the genome upstream of the corresponding attPV site. Primer pair attB_inErm_rev and attB2094_for demonstrated the integration of attN2 from pMM656 (line 3) and pMM656_*ldh*nP (line 4) into chromosomal gene BVU_2094. Integration of the attN2 site into the corresponding locus resulted in a PCR fragment size of 1021 bp. An integration of the plasmids into the attPV site located in BVU_2451 did not occur, since the addition of primers attB_inErm_rev and attB2451_for did not lead to a DNA fragment in the PCR assay (line 1 and 2).

```
PvLDH       MAYKIAFYDTKPYDERSFTEANEKFG-FDIRYYKGHLNMNNVVLTKGVDVVCIFVNDTAD      59
EcLDH       --MKLAVYSTKQYDKKYLQQVNESFG-FELEFFDFLLTEKTAKTANGCEAVCIFVNDDGS      57
PaLDH       --MRILFFSSQAYDSESFQASNHRHG-FELHFQQAHLQADTAVLAQGFEVVCAFVNDDLS      57
LcLDH       --MKIIAYGARVDEIQYFKQWAKDTG-NTLEYHTEFLDENTVEWAKGFDGINSLQTTPYA      57
LpLDH       --MKIIAYAVRDDERPFFDTWMKENPDVEVKLVPELLTEDNVDLAKGFDGADVYQQKDYT      58
LdLDH       -MTKIFAYAIREDEKPFLKEWEDAHKDVEVEYTDKLLTPETVALAKGADGVVVYQQLDYT      59
               ::  :  :  :   :    .      :.     *  ...  ::* :

PvLDH       AEVIRAMADNGVKLLALRCAGYNNVDLAAT-AGKMKVVRVPAYSPYAVAEFTVALMLSLN      118
EcLDH       RPVLEELKKHGVKYIALRCAGFNNVDLDAAKELGLKVVRVPAYDPEAVAEHAIGMMMTLN      117
PaLDH       RPVLERLAAGGTRLVALRSAGYNHVDLAAAEALGLPVVHVPAYSPHAVAEHAVGLILTLN      117
LcLDH       AGVFEKMHAYGIKFLTIRNVGTDNIDMTAMKQYGIRLSNVPAYSPAAIAEFALTDTLYLL      117
LpLDH       AEVLNKLADEGVKNISLRNVGVDNLDVPTVKARGLNISNVPAYSPNAIAELSVTQLMQLL      118
LdLDH       AETLQALADNGITKMSLRNVGVDNIDMAKAKELGFQITNVPVYSPNAIAEHAAIQAARIL      119
              .:. :   *   :::* .* :::*:       : : .**.*.* *:** :      :

PvLDH       RKIPRATMRTRDGNFSL-HGLMGFDMHGKTAGIIGTGKIAKILIQILRGFGMNVLAYDLY      177
EcLDH       RRIHRAYQRTRDANFSL-EGLTGFTMYGKTAGVIGTGKIGVAMLRILKGFGMRLLAFDPY      176
PaLDH       RRLHRAYNRTREGDFSL-HGLTGFDLHGKRVGVIGTGQIGETFARIMAGFGCELLAYDPY      176
LcLDH       RNMGKVQAQLQAGDYEKAGTFIGKELGQQTVGVMGTGHIGQVAIKLFKGFGAKVIAYDPY      177
LpLDH       RQTPMFNKKLAKQDFRW-APDIAKELNTMTVGVIGTGRIGRAAIDIFKGFGAKVIGYDVY      177
LdLDH       RQDKAMDEKVARHDLRW-APTIGREVRDQVVGVIGTGHIGQVFMQIMEGFGAKVIAYDIF      178
            *.      :    :        .  :    .*::***:*.     :: *** .::.:* :

PvLDH       PDYNFAREHQVVYCTLDELYHSSDIISLHCPLTEQTKYLINDYSISKMKDGVMIINTGRG      237
EcLDH       PSAAA-LELGVEYVDLPTLFSESDVISLHCPLTPENYHLLNEAAFEQMKNGVMIVNTSRG      235
PaLDH       PNPRI-QALGGRYLALDALLAESDIVSLHCPLTADTRHLIDAQRLATMKPGAMLINTGRG      235
LcLDH       PMKGD-HP-DFDYVSLEDLFKQSDVIDLHVPGIEQNTHIINEAAFNLMKPGAIVINTARP      235
LpLDH       RNAEL-EKEGMYVDTLDELYAQADVITLHVPALKDNYHMLNADAFSKMKDGAYILNFARG      236
LdLDH       RNPEL-EKKGYYVDSLDDLYKQADVISLHVPDVPANVHMINDESIAKMKQDVVIVNVSRG      237
                           *  *  .:*:: ** *    . ::::   :  ** .. ::* .*

PvLDH       QLIHTNALIEGLKTKKVGYAGLDVYEEEEPYFYEDKSDKIIDDDTLARLLSFNNVIVTSH      297
EcLDH       ALIDSQAAIEALKNQKIGSLGMDVYENERDLFFEDKSNDVIQDDVFRRLSACHNVLFTGH      295
PaLDH       ALVNAAALIEALKSGQLGYLGLDVYEEEADIFFEDRSDQPLQDDVLARLLSFPNVVVTAH      295
LcLDH       NLIDTQAMLSNLKSGKLAGVGIDTYEYETEDLLNLAKHGSFKDPLWDELLGMPNVVLSPH      295
LpLDH       TLIDSEDLIKALDSGKVAGAALVTYEYETKIFNKDLEGQTIDDKVFMNLFNRDNVLITPH      296
LdLDH       PLVDTDAVIRGLDSGKIFGYAMDVYEGEVGIFNEDWEGKEFPDARLADLIARPNVLVTPH      297
             *:.:   :  *.. ::   .: .** *   : :  .   : *     *    **:.: *

PvLDH       QAFFTKEAMTNIAHTTLQNVKDFAESRSLVNEVAVGRV  335
EcLDH       QAFLTAEALTSISQTTLQNLSNLEKGETCPNELV----  329
PaLDH       QAFLTREALAAIADTTLDNIAAWQDGTPRNRVRA----  329
LcLDH       IAYYTETAVHNMVYFSLQHLVDFLTKGETSTEVTGPAK  333
LpLDH       TAFYTETAVHNMVHVSMNSNKQFIETGKADTQVKFD--  332
LdLDH       TAFYTTHAVRNMVVKAFDNNLELVEGKEAETPVKVG--  333
             *: *  *:  :   :::
```

Fig. S2 Sequence alignment of representative D-LDHs. The amino acid sequences of the D-LDHs from *P. vulgatus* (PvLDH; A6L392), *P. aeruginosa* (PaLDH; Q9I530), *E. coli* (EcLDH; P52643), *L. delbrueckii*, (LdLDH; P26297) and *L. pentosus* (LpLDH; P26298) as well as the D-hydroxyisocaproate dehydrogenase from *Lactobacillus casei* (LcHDH; A0A0E2BVW2) were aligned using the program Clustal Omega (https://www.ebi.ac.uk/Tools/msa/clustalo/). The characteristic motifs for D-LDHs are marked according to Furukawa et al. (2018): Amino acids of the substrate-binding site are shown in red and the residues involved in the coenzyme binding are colored in blue. The two hinge regions between the catalytic and the NAD-binding domains are boxed.

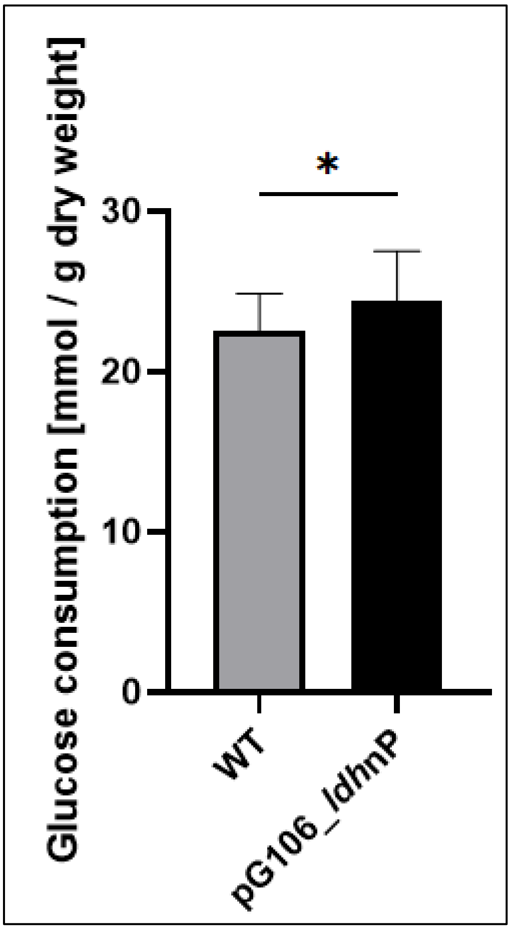

Fig. S3 Substrate consumption. *P. vulgatus* WT (gray) and *P. vulgatus* pG106_*ldh*nP (black) were grown in minimal medium with glucose as substrate. Cultures were harvested and the supernatants analyzed by HPLC. The amount of glucose consumption was correlated to the dry weight of the corresponding cultures. Values represent the average of at least 16 different cultures for each strain. For significance analysis data sets were analyzed by t-test using GraphPad Prism 8.0.2.263. * = $p \leq 0.05$.
